# Mouse Memory CD8 T Cell Subsets Defined by Tissue-Resident Memory Integrin Expression Exhibit Distinct Metabolic Profiles

**DOI:** 10.4049/immunohorizons.2300040

**Published:** 2023-10-19

**Authors:** Mike Sportiello, Alexis Poindexter, Emma C. Reilly, Adam Geber, Kris Lambert Emo, Taylor N. Jones, David J. Topham

**Affiliations:** * Center for Vaccine Biology and Immunology, University of Rochester Medical Center, Rochester, NY; † Medical Scientist Training Program, University of Rochester Medical Center, Rochester, NY

## Abstract

Tissue-resident memory CD8 T cells (T_RM_) principally reside in peripheral nonlymphoid tissues, such as lung and skin, and confer protection against a variety of illnesses ranging from infections to cancers. The functions of different memory CD8 T cell subsets have been linked with distinct metabolic pathways and differ from other CD8 T cell subsets. For example, skin-derived memory T cells undergo fatty acid oxidation and oxidative phosphorylation to a greater degree than circulating memory and naive cells. Lung T_RM_s defined by the cell-surface expression of integrins exist as distinct subsets that differ in gene expression and function. We hypothesize that T_RM_ subsets with different integrin profiles will use unique metabolic programs. To test this, differential expression and pathway analysis were conducted on RNA sequencing datasets from mouse lung T_RM_s yielding significant differences related to metabolism. Next, metabolic models were constructed, and the predictions were interrogated using functional metabolite uptake assays. The levels of oxidative phosphorylation, mitochondrial mass, and neutral lipids were measured. Furthermore, to investigate the potential relationships to T_RM_ development, T cell differentiation studies were conducted in vitro with varying concentrations of metabolites. These demonstrated that lipid conditions impact T cell survival, and that glucose concentration impacts the expression of canonical T_RM_ marker CD49a, with no effect on central memory-like T cell marker CCR7. In summary, it is demonstrated that mouse resident memory T cell subsets defined by integrin expression in the lung have unique metabolic profiles, and that nutrient abundance can alter differentiation.

## Introduction

Tissue-resident memory CD8 T cells (T_RM_) and tumor-infiltrating lymphocytes (TILs) with a T_RM_-like phenotype (CD49a^+^, CD103^+^) (integrin α1^+^, integrin αEβ7^+^) confer protection against a variety of illnesses ranging from infectious diseases to cancers. As demonstrated in the skin, transfer of T_RM_s from immune to naive hosts is sufficient to prevent HSV type 1 (HSV-1)-associated pathology, and comparably, the presence of Ag-specific CD49a^+^CD103^+^ TILs in a mouse model of melanoma can limit tumor growth (1, 2). In the lungs, T_RM_s are critical for protection against SARS-CoV-2 (COVID-19), influenza, and respiratory syncytial virus, even in the absence of Abs specific for those viruses (3–6). T_RM_s have been shown to act both as Ag-specific sentinels linking the innate and adaptive immune systems and as cytotoxic mediators (7). This functional capacity makes them important players in immunity across multiple organ systems, particularly barrier tissues where nutrient substrate availability varies, and disease states.

T_RM_s are heterogeneous in terms of integrin expression, migration, effector potential, and gene expression (7, 8). Furthermore, the functions of different memory CD8 T cell subsets have been linked with distinct metabolic pathways. For example, skin T_RM_s undergo fatty acid oxidation to a greater degree than other circulating memory and naive cells, which tend to have higher glycolytic fluxes (9, 10). However, it is unclear whether this programming is universal among all memory T cell subsets and whether it holds true in other tissues such as the lung. It is also not yet known whether T_RM_ CD8 T cell subsets that express different combinations of surface integrins CD49a and CD103 share metabolic profiles. RNA sequencing (RNAseq) on these populations revealed distinct metabolic transcriptomes (7), suggesting the overall metabolic profiles may differ, which was supported by ex vivo assays. In vitro studies to drive differentiation into T_RM_-like phenotypes were used to test whether manipulating nutrient availability affected integrin-based subset development.

## Materials and Methods

### RNAseq analysis

RNAseq data and lists of differentially expressed genes were obtained from previously published work from our laboratory (GSE179653). Full code that generated those differentially expressed genes, as well as further analyses and figures, is publicly available at https://github.com/sportiellomike/Immunohorizons-2023-Lung-CD8-Metabolism.

### Metabolic modeling

Mouse metabolic model iMM1865 was used as a starting point, onto which our publicly accessible transcriptomic data for CD49a^+^CD103^+^ (double positive [DP]), CD49a^+^CD103^−^ (CD49a single positive [SP]), or CD49a^−^CD103^−^ (double negative [DN]) were applied using the Gene Inactivity Moderated by Metabolism and Expression (GIMME) algorithm (11). A threshold value of the median transcript per kilobase million for the 1865 genes in the metabolic model was used. Reaction constraints were added according to previously published research (12). All code used to generate input data, figures, and the models themselves is available at https://github.com/sportiellomike/Immunohorizons-2023-Lung-CD8-Metabolism.

### Mice

Mice were housed in approved microisolator cages, within a pathogen-free vivarium facility that is accredited by the Association for Assessment and Accreditation of Laboratory Animal Care. The vivarium is staffed with a number of personnel, including those with expertise in husbandry, technical skills, and veterinary personnel. C57BL/6J mice (Jackson Laboratories) used for experiments were infected 8–10 wk after birth. This study was carried out in strict accordance with the recommendations in the *Guide for the Care and Use of Laboratory Animals* as defined by the National Institutes of Health (NIH). The Institutional Animal Care and Use Committee of the University of Rochester approved all protocols.

### Virus and infection

Mice were anesthetized with 3,3,3-tribromoethanol. Upon verification of sedation, mice were infected intranasally with 10^5^ EID_50_ (50% embryo infectious dose) of HKx31 human influenza A virus in 30 μl volume. After observation to ensure they recovered from anesthesia, mice were monitored daily for weight and overall morbidity to comply with all regulations.

### Lung and bronchoalveolar lavage processing

Bronchoalveolar lavage (BAL) was collected by inserting a flexible Teflon catheter into a slit in the trachea in proximity to the larynx. A 1-ml syringe was connected to the catheter, and the lungs were flushed three times with 1× PBS (volumes of 1 ml, 900 μl, and 800 μl). BAL was spun down at 424 × *g* for 10 min. RBCs were eliminated through ACK-mediated lysis. Cells were washed in 1× PBS containing 1% FBS (PBS+serum) and resuspended in PBS+serum.

The lungs were harvested and separated into right and left lobes before collection into RPMI + 8% FBS (10040CV; Corning). Lungs were transferred into Miltenyi gentleMACS tubes containing 2 ml of 2 mg/ml collagenase II (LS004177; Worthington) and mechanically ground up by a gentleMACS machine. Three additional milliliters of 2 mg/ml collagenase II solution was added before incubation for 30 min at 37°C. Further mechanical digestion by gentleMACS was done at this point. Tubes were spun at 233 × *g* at room temperature for 2 min.

Pellets were then dissolved in existing supernatant and transferred through 100-µm strainers. Contents were quantitatively transferred using 5 ml of RPMI + 8% FBS. Tubes were spun at 424 × *g* for 6 min at room temperature. The supernatant was discarded and dissolved in 4 ml 40% Percoll solution, which was then layered on 3 ml 75% Percoll solution. These tubes were then spun at 931 × *g* for 20 min at room temperature with no centrifuge break so as not to disturb the layers of the gradient.

The top layer of lipid-rich liquid was aspirated off, and the interface (∼2 ml) of the two Percoll densities was collected where the cells were. Tubes were filled with RPMI + 8% FBS and spun down at 424 × *g* for 6 min at room temperature.

### Spleen processing to achieve single-cell suspension

C57BL/6J mouse spleens were obtained from sacrificed mice and mechanically digested by grinding between two frosted microscope slides without exogenous enzyme added. After grinding, single-cell suspensions were passed through a plastic mesh to capture large nonsingle cellular material and centrifuged in conical vials at 424 × *g* at 4°C for 6 min. Cells were resuspended in 3 ml of ACK lysis buffer for 5 min at room temperature. Volume was brought up to 15 ml in PBS + serum followed by centrifuging at 424 × *g* at 4°C for 6 min.

### T_RM_-like media preparation

The following were added to 500 ml DMEM (Catalog No. [Cat.] 10-017-CV; Corning): 50 ml FBS, 5 ml l-glutamine (Cat. 25030081; Fisher Scientific), 12.5 ml HEPES buffer (Cat. 15630080; Fisher Scientific), 6.5 MEM Non-Essential Amino Acids Solution (Cat. 11140050; Fisher Scientific), 0.6 ml of 1:300 dilution of 2-ME in PBS, 5 ml penicillin-streptomycin (Cat. 15140122; Fisher Scientific), and 0.6 ml gentamicin (Cat. 15710064; Fisher Scientific). Contents were sterile filtered.

### Fatty acid uptake

Fatty acid uptake media were created by dissolving 2 µl stock solution made according to package insert per 1 ml of Assay Buffer (Screen Quest Fluorimetric Fatty Acid Uptake Assay Kit 36385; AAT Bioquest). Once a single-cell suspension was obtained from the harvesting protocol, cells were plated in 100 µl of fatty acid uptake media and 100 µl Leibowitz media for a final volume of 200 µl. Vehicle control used equivalent volume of DMSO in Assay Buffer. Cells were incubated for 30 min at 37°C, then washed 1.5 times with PBS + 1% serum before staining.

### Neutral lipid staining

Bodipy 505/515 (Cat. D3921; Fisher Scientific) was dissolved in DMSO to yield a stock of 1 mg/µl concentration. Once a single-cell suspension was obtained from the harvesting protocol, each sample of cells in 1 ml of PBS was inoculated with Bodipy to yield a final concentration of 2 mg/ml or DMSO for the vehicle control. Samples were incubated for 30 min at 37°C, then washed 1.5 times with PBS + 1% serum before staining.

### 2-(*N*-(7-nitrobenz-2-oxa-1,3-diazol-4-yl)amino)-2-deoxyglucose uptake

2-(*N*-(7-nitrobenz-2-oxa-1,3-diazol-4-yl)amino)-2-deoxyglucose (2-NBDG) (Cat. ab146200; Abcam) was dissolved in DMSO to yield a 20 mM stock solution. After a single-cell suspension was obtained from the harvesting protocol, cells were resuspended in 500 µl nonsupplemented Leibowitz media and incubated at 37°C with a final 2-NBDG concentration of 80 µM or DMSO for the vehicle control for 35 min at 37°C. Samples were washed 1.5 times with PBS + 1% serum before staining.

### Mitochondrial mass quantification

MitoTracker Green FM (MitoTracker) (Cat. M7514; Fisher Scientific) was dissolved in DMSO to obtain a 1 mM stock solution. After a single-cell suspension was obtained from the harvesting protocol, each sample was dissolved in 500 µL PBS + 1% serum. Samples were inoculated with MitoTracker for a final concentration of 50 nM or equivalent volume of DMSO for the vehicle control and incubated for 45 min at 37°C.

### Oxidative phosphorylation quantification

Tetramethylrhodamine, ethyl ester, perchlorate (TMRE) (Cat. 701310; Cayman Chemical) was prepared in a 0.5 mM working stock by dissolving in DMSO, according to package insert. After a single-cell suspension was obtained from the harvesting protocol, TMRE was added to two separate aliquots of 5 ml RPMI + 8% FBS (TMRE media) for a final concentration of 250 nM. To one of these aliquots, 2-[2-[4-(trifluoromethoxy)phenyl]hydrazinylidene]-propanedinitrile (FCCP) was added (FCCP-TMRE media) for a final concentration of 1 µM. Samples were split into two and dissolved into 500 µl of either TMRE media or FCCP-TMRE media. Samples were incubated at 37°C for 30 min. For vehicle control, DMSO was added in place of TMRE. Cells were washed 1.5 times with PBS + 1% FBS before staining.

### T_RM_-like cell culture conditions

Twenty-four-well plates were coated with anti-CD3/28–stimulating Abs (Cat. 100302 and 102102; BioLegend) at final concentration of 5 µg/ml each overnight at 4°C. The next day, 2 million single-cell splenocytes were plated in 1 ml T_RM_-like culture media onto each well with 7.5 ng/ml human IL-2 (obtained from the NIH). Two days later, cells were collected, centrifuged at 424 × *g* at 4°C for 6 min, and plated onto noncoated 24-well plates in 1 ml T_RM_-like culture media with 7.5 ng/ml IL-2 for 3 d. After that, cells were collected, centrifuged at 424 × *g* at 4°C for 6 min, and plated in T_RM_-like culture media with 10 ng/ml mouse IL-7 (Cat. 577804; BioLegend) and 10 ng/ml human TGF-β1 (PHG9214; Fisher Scientific) for 6 d.

Additional glucose (Cat. A2494001; Fisher Scientific) and lipid supplement (Cat. L5146; Millipore Sigma) were added to T_RM_-like cell culture media starting from the day of spleen harvest (day 0) throughout the end of the experiment (day 11, day 6 after TGF-β1 addition). Soluble lipid stores were created in Kolliphor P 188 (K4894; Millipore Sigma) according to lipid supplement instructions.

### Staining for flow cytometry

Cells were suspended in 100 µl staining buffer for 30 min at 4°C. Staining buffer was created by adding staining Abs to PBS below for a final concentration of 1 µg/ml each, or 0.2 µl of live-dead stock solution/100 µl staining buffer. Live-dead stock solution was prepared according to package insert available on the vendor Web site. Anti-CD16/32 was added for a final concentration of 2.5 µg/ml. Anti-CD45 was previously injected at time of organ harvest i.v. at a concentration of 0.2 µg stock Ab/100 µl PBS in a volume of 100 µl. All Abs used Bangs Laboratories compensation beads (556), which were stained an equivalent time. Live-dead stain used Life Technologies ArC reactive beads (A10346; Invitrogen). Because the vast majority of cells at day 14 postinfection will have been Ag experienced, CD44 was not used as a marker on day 14 experiments. Experiments were run on an 18-color BD LSR II flow cytometer. Gating strategies are displayed in Supplemental Figs. 1 and 3B. For staining, we used live-dead aqua (Cat. L34966) and CD8α-allophycocyanin-eFluor 780 (Cat. 47-0081-82) from Invitrogen. The following were used from BioLegend: i.v. CD45-BV785 (Cat. 103149), CD8α-allophycocyanin (Cat. 100712), TCRβ-BV650 (Cat. 109251), CD103-BV421 (Cat. 121422), CD44-allophycocyanin (Cat. 103012), CCR7-PE-Dazzle 594 (Cat. 120122), and Fc Block anti-mouse (anti-CD16/32) (Cat. 101302). The following were used from BD Biosciences: CD49a-PE (Cat. 562115) and CD49a-BV711 (Cat. 562115).

### Median fluorescence intensity determination and normalization

The four subsets of interest were manually gated after selecting for lymphocytes (forward light scatter [FSC]-A and side scatter-A), singlets (FSC-A and FSC-H), live cells, IV-CD45^−^ cells, and TCRβ^high^ and CD8α^high^ cells. DN was defined as CD103^low^ and CD49a^low^, DP as CD103^high^ and CD49a^high^, CD103 SP as CD103^high^ and CD49a^low^, and CD49a SP as CD49a^high^ and CD103^low^.

Median fluorescence intensities (MFIs) were calculated using FlowJo (version 10.6.2). Data were normalized by dividing the MFI of each subset by the sum of all four subsets’ MFIs for each mouse and multiplying by 4. For the assays using TMRE, FCCP was subtracted from the TMRE MFI for each subset within each mouse. That number was divided by the sum of MFIs of the uncoupled control (FCCP+TMRE) subsets for each mouse. Normalized data can be found online at https://github.com/sportiellomike/Immunohorizons-2023-Lung-CD8-Metabolism.

### Statistical tests

After repeated-measures ANOVA, multiple pairwise comparisons were evaluated using the Student *t* test with Benjamini-Hochberg correction. If Adjusted *p* value (*p*_adj_) is <0.05, results were deemed significant. R Packages ggplot2 (version 3.3.2) and rstatix (0.7.0) were used for data plotting and statistical testing, respectively. The code used to perform statistical tests and generate figures can be found online at: https://github.com/sportiellomike/Immunohorizons-2023-Lung-CD8-Metabolism.

### Ethics statement

This study was carried out in strict accordance with the recommendations in the *Guide for the Care and Use of Laboratory Animals* as defined by the NIH. Animal protocols were reviewed and approved by the Institutional Animal Care and Use Committee of the University of Rochester in writing. All animals were housed in a centralized and Association for Assessment and Accreditation of Laboratory Animal Care–accredited research animal facility that is fully staffed with trained husbandry, technical, and veterinary personnel. Our University Committee on Animal Resources number is 2006-029.

Euthanasia was performed by animal welfare committee review–approved methods.

### Data availability

The datasets for this study can be found on GitHub (https://github.com/sportiellomike/Immunohorizons-2023-Lung-CD8-Metabolism). Previously published RNAseq datasets were used and are available via GEO accession number GSE179653 (https://www.ncbi.nlm.nih.gov/geo/query/acc.cgi?acc=GSE179653).

## Results

### Pathway analysis for metabolic analyses

T_RM_s can be defined by their surface protein expression, with multiple markers having been used, including CD69, CD49a, and CD103 (13). In the mouse lung, CD44 expression identifies Ag-experienced memory T cells, whereas CD49a marks T_RM_s, and when used in combination with CD103, CD49a yields two T_RM_ subsets: CD49a^+^CD103^+^ (DP) and CD49a^+^CD103^−^ (CD49a SP) (7). At later memory time points, very few CD49a^−^CD103^+^ (CD103 SP) cells remain, suggesting they have either died, left the tissue, or altered their integrin expression. CD49a^−^CD103^−^ (DN) cells represent mixed phenotypes, although more than half are CD44^high^CD62L^low^ T cells, consistent with an effector memory profile, and may be circulating (7). To more fully describe the metabolic profiles of T_RM_s based on integrin expression, we used the publicly accessible RNAseq dataset we previously reported (7). This dataset was generated from CD44^high^ mouse lung tissue CD8 T cells 21 d postinfection (dpi) that were sorted by CD49a and CD103 expression.

**FIGURE 1.**
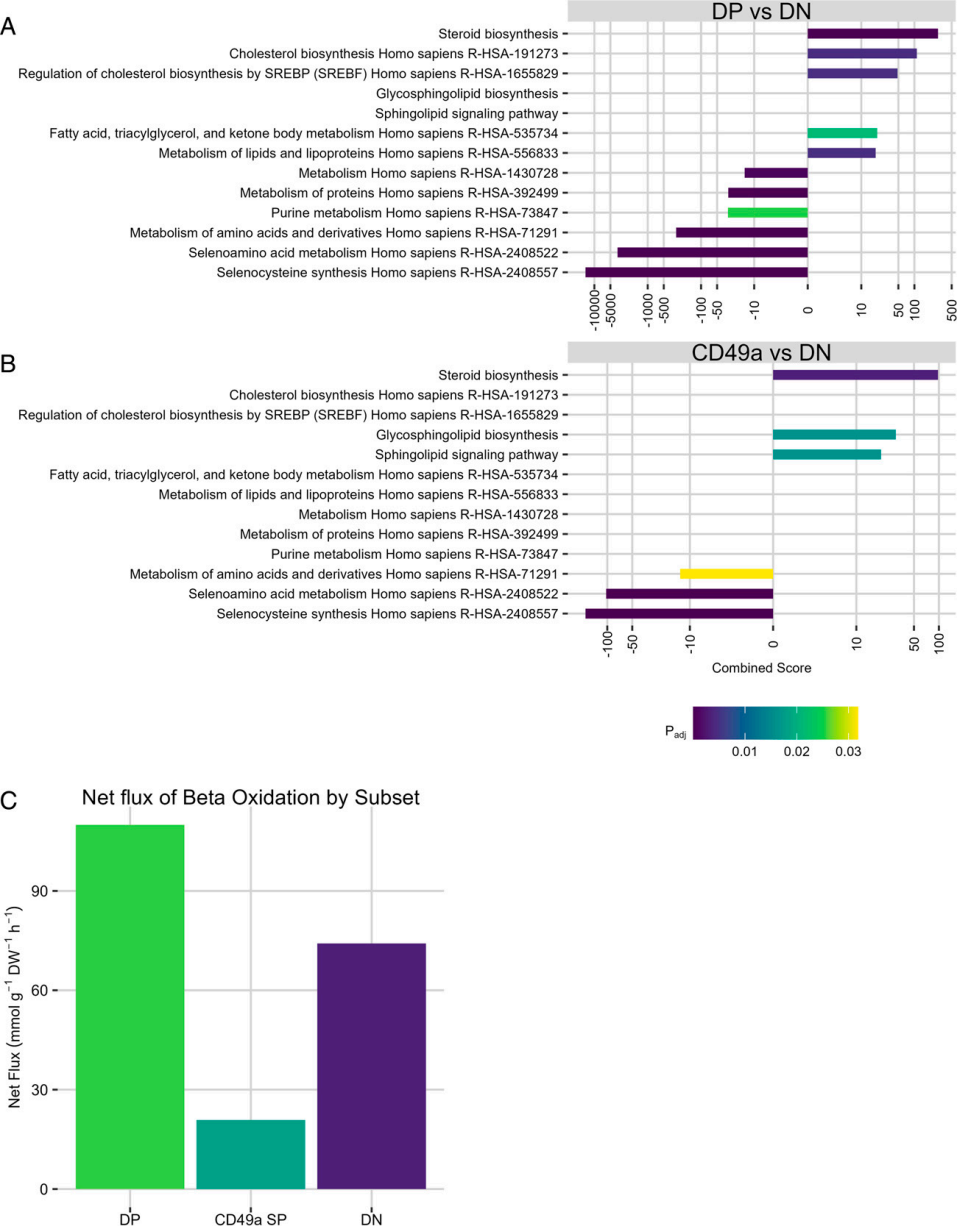
Transcriptomic analysis of lung CD8 T cells 21 dpi. Reactome and KEGG databases were queried with the lists of differentially expressed genes between (**A**) DP and DN, or (**B**) CD49a SP and DN. Criteria for inclusion as differentially expressed genes include *p*_adj_ < 0.05 and Log_2_(fold change) > 0.5 or Log_2_(fold change) < 0.5. *p*_adj_ cutoff for enrichment is *p*_adj_ < 0.05. Only select pathways are shown. A complete list can be found in the supplemental information. Datasets were generated using 10 total mice pooled and sequenced per independent experiment with three pools of those 10 mice per subset. Metabolic models were created using the GIMME algorithm, and the level of flux through β oxidation was plotted (**C**). CD49a SP, CD49a^+^CD103^−^; DN, CD49a^−^CD103^−^; DP, CD49a^+^ CD103^+^.

Using *p*_adj_ < 0.05 as a cutoff, the data were analyzed to identify genes with a minimum of Log_2_(*x*) = 0.5 fold change to determine upregulated or downregulated genes, respectively. Per pathway analysis using both the Reactome and Kyoto Encyclopedia of Genes and Genomes (KEGG) databases, there were no significant differences when comparing DP with CD49a SP. However, several metabolic pathways were upregulated in DP versus DN, including *Metabolism of lipids and lipoproteins*, *Fatty acid, triacylglycerol, and ketone body metabolism*, and *Cholesterol biosynthesis*. A selection of pathways is plotted in Fig. 1A and 1B. The complete set of significantly enriched pathways can be found in Supplemental Table I.

Interestingly, CD49a SP and DP did not share any strictly metabolic pathways from the Reactome when compared with DN, yet they were indistinguishable from one another at the global transcriptomic level (7) (Fig. 1B). They also did not share any pathways with each other in the KEGG database beyond *Steroid biosynthesis* (Fig. 1B). Metabolic differences between DP and DN, but not CD49a SP and DN, could imply metabolic differences between CD49a SP and DP that these pathway analysis methods are not sensitive enough to distinguish. In summary, T_RM_ subsets defined by integrin expression were metabolically distinguishable from DN cells based on gene expression.

### Metabolic modeling predicts lipid-centric metabolism in T_RM_s

Having performed basic pathway analyses and discovering a possible enrichment for lipid-centric programming in T_RM_s, we developed an approach for modeling the metabolome of lung CD8 T cells using the entirety of the transcriptomic data and used this more intensive approach to verify the results of the basic analysis.

Metabolic models are networks of metabolites and the reactions that use and produce them. Generally, these reactions are catalyzed using genome-encoded enzymes. Three metabolic computational models were constructed: one for lung CD8 T cells expressing both CD49a and CD103 (*tissuemodelDP*), one for the SP subset (*tissuemodelCD49aSP*), and one for the subset expressing neither integrin (*tissuemodelDN*). With these models, we used the expression of enzymes present in a system to predict the amount of each metabolite present. These models together incorporate nearly all Ag-experienced CD8 T cells in mouse lungs, encompassing both T_RM_s and non-T_RM_ memory T cells.

In brief, the published mouse metabolic model iMM1865 was used as a starting point (14). Then, the GIMME algorithm was implemented in the COBRA Toolbox (15) to conduct a flux balance analysis using our RNAseq transcript-level data to inform the level of flux for each reaction (11). GIMME alters the level of flux through a reaction to minimize the total inconsistency score. Assuming a larger quantity of enzyme should produce a larger flux through that reaction, GIMME increases (or decreases) flux through different reactions depending on the level of transcript for that enzyme. When building metabolic models, an objective function must be selected for which flux is to be maximized given the constraints. In line with most other metabolic models, “BIOMASS” was selected, which itself is a combination of several reactions related to cell maintenance that must have positive flux for cells to be viable. Given our constraints, a single, nonzero optimal solution was predicted for all three of our models.

Our model predicted that our DP subset had the greatest flux through its β *Oxidation of Fatty Acids* pathway, consistent with our initial pathway analysis (Fig. 1C). In addition, the CD49a SP subset differed from the DP subset with less flux through that pathway than both DP and DN, again consistent with the initial pathway analysis. These results, in addition to our basic pathway analysis, imply differences in T_RM_ metabolism between DP and CD49a SP subsets, as well as between integrin-expressing and DN subsets.

### T_RM_ CD8 T cell subsets have unique metabolic profiles directly ex vivo

Thus far, our transcriptomic analyses were largely consistent with that demonstrated for bulk T_RM_s in the skin (10). Nonetheless, transcriptomic data and metabolic modeling do not clearly demonstrate that functional differences exist between cell subsets whether circulating or tissue memory. The Seahorse XF Mito Fuel Flex assay, which (ideally) requires large cell numbers, is considered a gold standard for measuring a number of metabolic metrics such as oxygen consumption and acidification rate. However, because of limiting cell numbers and our interest in the resting (not restimulated) metabolic state of memory cells, the signal was not high and reproducible enough to draw conclusions, demonstrating this approach is not feasible with unstimulated primary memory T cells. Instead, to capture single-cell data, we performed a flow-cytometric approach. T cells with T_RM_-like integrin expression are evident in mouse lung T cells 14 d dpi with HKx31 influenza A virus and may include cells with memory potential, whereas by 60 dpi memory is well established. To determine whether the T cell subsets display any differences in their mitochondria, we evaluated the mitochondrial mass of cells of different integrin profiles by MitoTracker Green FM (MitoTracker) staining. MitoTracker preferentially accumulates in mitochondria by covalently binding free thiol groups independent of mitochondrial membrane potential (16). Both airway cells harvested through BAL and cells remaining in the tissue after lavage (likely lung parenchymal cells) were examined for MitoTracker staining. Tissue vasculature-associated cells were excluded using a standard i.v. labeling approach (7).

**FIGURE 2.**
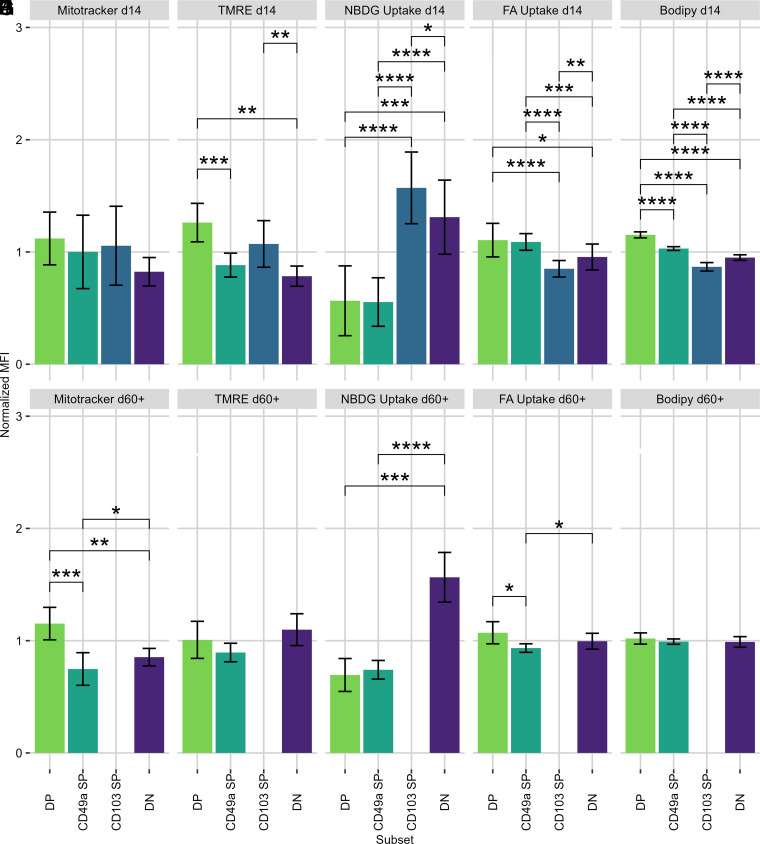
Normalized mouse IV-label–negative lung CD8 T cell MFIs from flow-cytometric assays. Normalized MFIs of IV-label–negative lung CD8 T cells plotted for each assay at 14 and 60+ dpi derived from flow-cytometric analysis. Mitochondrial mass was assessed with MitoTracker Green FM (**A** and **B**). Mitochondrial membrane potential was assessed with TMRE (**C** and **D**). Glucose uptake was assessed with glucose analogue 2-NBDG (**E** and **F**). Fatty acid uptake was assessed with fluorescently conjugated fatty acids (**G** and **H**). Neutral lipid stores were assessed with Bodipy (**I** and **J**). Data generated from two independent experiments of *n* = 4–10 mice per experiment. CD49a SP, CD49a^+^CD103^−^; CD103 SP, CD49a^−^CD103^+^; DN, CD49a^−^CD103^−^; DP, CD49a^+^CD103^+^. *p < 0.05, **p < 0.01, ***p < 0.001, ****p < 0.0001.

We found no differences in MitoTracker staining in the lung or BAL at day 14 (Fig. 2A, Supplemental Fig. 2). However, at day 60 dpi, CD49a SP had significantly lower levels of mitochondrial mass than DP and DN counterparts in the lung, whereas DP had the highest staining (Fig. 2B). This suggests that as memory T cell subsets develop, mitochondrial mass changes.

The question remained whether the cell populations displayed differential mitochondrial functions, not just different mitochondrial masses. Oxidative phosphorylation is a primary role of the mitochondrion, because it is the most efficient means by which a cell can generate ATP (17). Cells with equivalent mitochondrial mass could potentially undergo differential rates of oxidative phosphorylation and therefore ATP production. Importantly, cells could be undergoing different levels of oxidative phosphorylation but be producing similar amounts of ATP overall through other mechanisms. Oxidative phosphorylation is linked to mitochondrial membrane polarization. TMRE, a fluorescent dye that preferentially stains polarized mitochondrial membranes, was used as a proxy for processes that polarize the mitochondrion, although TMRE is not a direct readout of oxidative phosphorylation. To remove background staining, we treated an equivalent sample with both TMRE and the decoupling agent FCCP, whose signal was subtracted from the treatment group’s signal.

Cells in the lung expressing CD103 had increased TMRE staining, implying a greater degree of membrane polarization and therefore oxidative phosphorylation 14 dpi (Fig. 2C). Later, at 60+ dpi, no subset was significantly different (Fig. 2D). At 60+ dpi, DP and DN cells have more mitochondrial mass than CD49a SP cells (Fig. 2B) but have the same membrane potential (Fig. 2D). Although not the only possibility, this is consistent with cells that have a higher spare respiratory capacity (SRC), i.e., they may have the capability to use their higher amounts of mitochondria to produce more ATP after reactivation.

Based on the observed differences, we wanted to investigate whether nutrient uptake was altered in a similar way. Glucose is a metabolically essential molecule in a variety of cellular processes as both a source of cell energy in the form of ATP, as well as a carbon source in anabolism (18). To evaluate the extent to which glucose was being taken up by the CD8 T cells, we used the glucose analogue 2-NBDG. We thought this a more sensitive readout of actual cell function than our pathway analysis in Fig. 1, which did not show significant differences in glucose import or glycolysis. This fluorescent analogue undergoes facilitated diffusion through glucose transporters GLUT1–4, as well as through active transport through SGLT1 and SGLT2, recapitulating the kinetics of glucose transport (19–24). It is phosphorylated by hexokinase after entering the cell, which traps the fluorescent molecule in the cell in a form that cannot proceed further in glycolysis.

Lung DP and CD49a SP displayed similar levels of 2-NBDG staining 14 dpi; however, these populations displayed lower levels of staining compared with DN and CD103 SP cells, suggesting that CD49a-expressing subsets may be less glucose dependent than CD49a^−^ (non-T_RM_) counterparts (Fig. 2E). A similar pattern was observed in BAL samples, with CD49a^+^ subsets taking up lower levels of the dye (Supplemental Fig. 2). This pattern persisted at later memory time points 60+ dpi (Fig. 2F) and is consistent with studies done on T_RM_s in the skin, which noted an increased dependence on free fatty acids (FFAs) as opposed to glucose (10).

Having demonstrated significant differences in the uptake of the glucose analogue 2-NBDG, we next investigated the ability of T cells to take up FFAs with the hypothesis that lung T_RM_s use a more lipid-centric metabolism, similar to skin (10). A fluorescently conjugated fatty acid (FFA) was used to measure uptake. In the lung parenchyma, CD49a^+^ subsets (CD49a SP and DP) took up the highest amount of FFA (Fig. 2G). Similar results were found among the BAL subsets. These findings were similar but distinct at 60+ dpi: DP and DN took up more FFAs than CD49a SP (Fig. 2H). Other studies that have reported a dependence of T_RM_s on FFAs have not used CD49a as a marker (10) and therefore may have excluded the CD49a^+^CD103^−^ subset while possibly including the CD103 SP subset. We expected our results to demonstrate that both DP and CD49a SP (the latter not studied by other metabolic studies) were equally dependent on these FFAs in the lung, but we found this to be true only at 14 dpi. At 60+ dpi, DP subsets were much more like DN than their CD49a SP T_RM_ counterparts. One interpretation consistent with the data is that CD49a SP uses a less lipid-centric metabolic strategy than its DP counterparts.

We next wanted to determine whether there were further differences in metabolic lipids. To address this, we used Bodipy, a molecule that is taken up passively and stains cellular stores of neutral lipids, to quantify the amount of neutral lipid present in lung T cells (25). Neutral lipids serve as a store of potential energy and as a carbon source, a valuable resource if the cells were to expand and undergo effector functions in a nutrient-deficient environment (26, 27).

Nearly identical patterns for BAL and lung were observed at 14 dpi (Fig. 2I, Supplemental Fig. 2). DP cells displayed the highest levels of neutral lipid storage, followed by CD49a SP, then DN, with the lowest levels in CD103 SP. Although all six comparisons demonstrate that the four subsets are significantly different from one another, it is remarkable that the subsets expressing CD49a (CD49a SP and DP) stain brightly for neutral lipids, whereas those not expressing CD49a (CD103 SP and DN) display low levels. Having quick access to neutral lipids for both energy and carbon needs may prove important for their protective function and/or long-term memory formation given these differences observed at 14 dpi were not seen at 60+ dpi (Fig. 2J).

### Glucose and lipid metabolites alter the phenotype of T_RM_-like cells in vitro

Noting the myriad of differences between T_RM_s and non-T_RM_s, as well as among T_RM_ subsets, we hypothesized that the metabolic microenvironment is deterministic in their differentiation. Given that tissue-specific metabolic substrates are impossible to control, an in vitro approach was employed. T_RM_ differentiation in vivo requires TGF-β (28). Similarly, T cells with T_RM_-like integrin profiles can be generated by stimulating naive mouse splenocytes under defined conditions that include TGF-β (29). In brief, T cells were initially activated by culture on anti-CD3/CD28-coated plates for 2 d in the presence of IL-2, followed by 3 d in noncoated plates supplemented with IL-2. T_RM_-like differentiation was achieved by placing these cells in IL-7 and TGF-β for 6 more days to elicit a T_RM_-like surface phenotype (29). To test the effects of glucose or lipids within and across levels, both the concentrations of glucose and of lipids were independently varied (Supplemental Fig. 3A). Glucose concentrations began with the concentration of glucose in standard cell culture media (4.5 mg/ml). This concentration has been demonstrated to ensure optimal T cell viability and activation, especially over the course of several days in culture because glucose is rapidly used by cells, although it is higher than that found in the blood or most other tissue microenvironments. To assess the impact of lipids on T cell differentiation in vitro, we used a broad range of exogenous lipid concentrations, which include both fatty acids and cholesterol (L5146; Millipore Sigma). Lipid supplementation varied from no exogenous lipid to 8× the concentration often used for cell culture.

**FIGURE 3.**
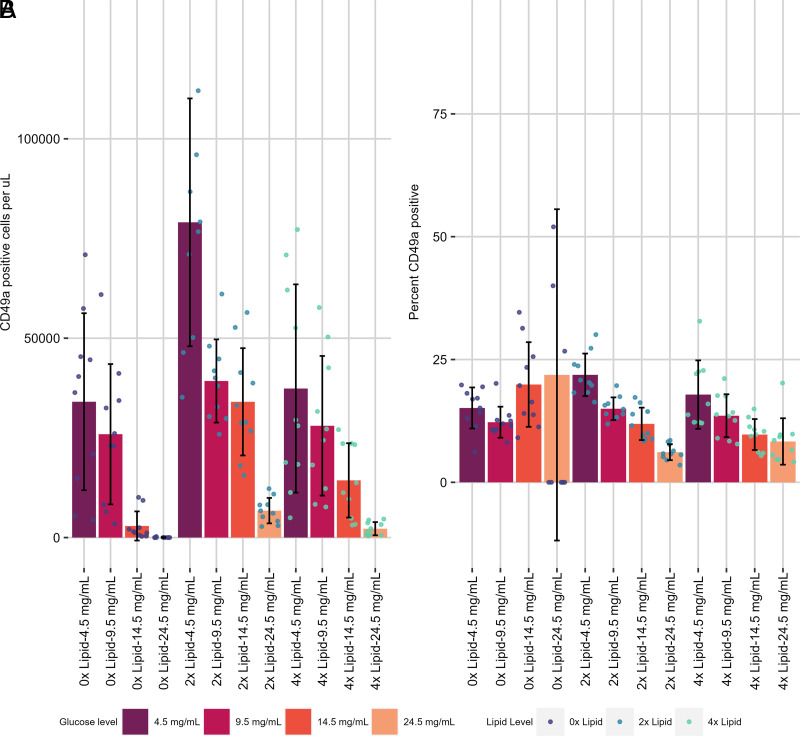
Metabolite concentration impact on T_RM_-like cell differentiation and maintenance in vitro. CD49a^+^ cells per microliter (**A**) and percent CD49a^+^ (**B**) were assessed in metabolite alteration assays where both lipid (0×–4×) and glucose (4.5–24.5 mg/mL) were altered after activation in the presence of IL-2 and cultured with IL-7 and TGF-β. Small circles represent individual data points. Data generated from two independent experiments of *n* = 5 mice per experiment.

**FIGURE 4.**
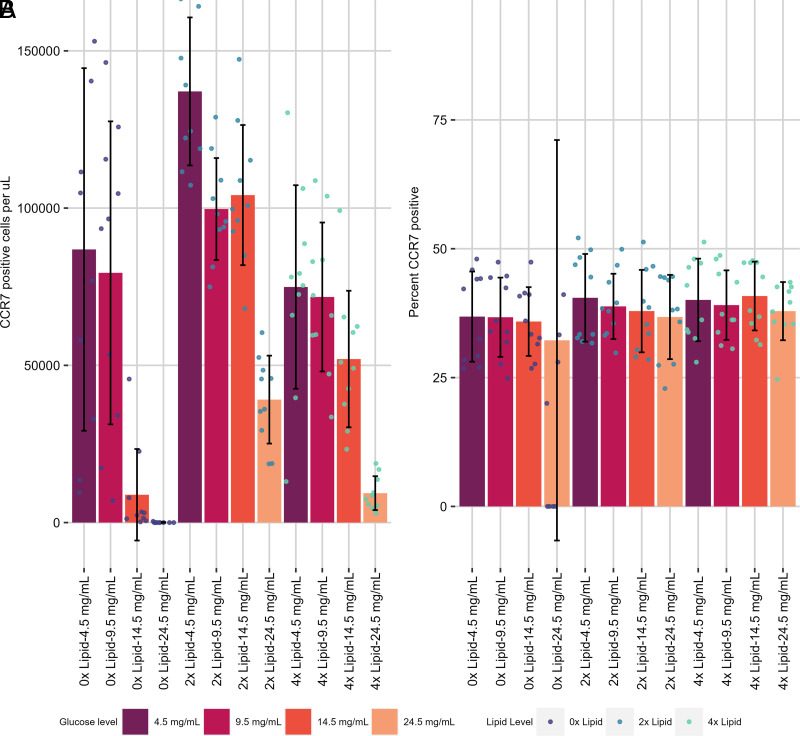
Metabolite concentration impact on T_CM_-like cell differentiation and maintenance in vitro. CCR7^+^ cells per microliter (**A**) and percent CD49a^+^ (**B**) were assessed in metabolite alteration assays where both lipid (0×–8×) and glucose (4.5–24.5 mg/ml) were altered after activation in the presence of IL-2 and cultured with IL-7 and TGF-β. Small circles represent individual data points. Data were generated from two independent experiments of *n* = 5 mice per experiment.

First, the effect on CD49a expression was examined. Without exogenous lipid added, there was no effect of glucose concentration on frequency, but with 2× or 4× lipid supplementation, generally, as the concentration of glucose increased, the cell number (normalized per volume) and proportion of CD49a^+^ cells both decreased (Fig. 3A, 3B, Supplemental Fig. 3, Table I). The relationship between the absolute number of CD49a^+^ cells per volume and glucose concentration was linear. Lipid supplementation (8×) was toxic because the number of live CD8 T cells for the group was effectively zero, so this group was removed from future analysis (Supplemental Fig. 4). Overall, this paints a picture of increasing glucose concentration causing a decrease in both the frequency and the absolute number of CD49a^+^ cells. This contrasts with the frequency of central memory marker CCR7 positivity (Fig. 4A, 4B, Supplemental Fig. 3, Table I), which did not vary with lipid supplementation, although increasing concentrations of glucose did seem to decrease the frequency of CCR7^+^ cells.

**Table:**
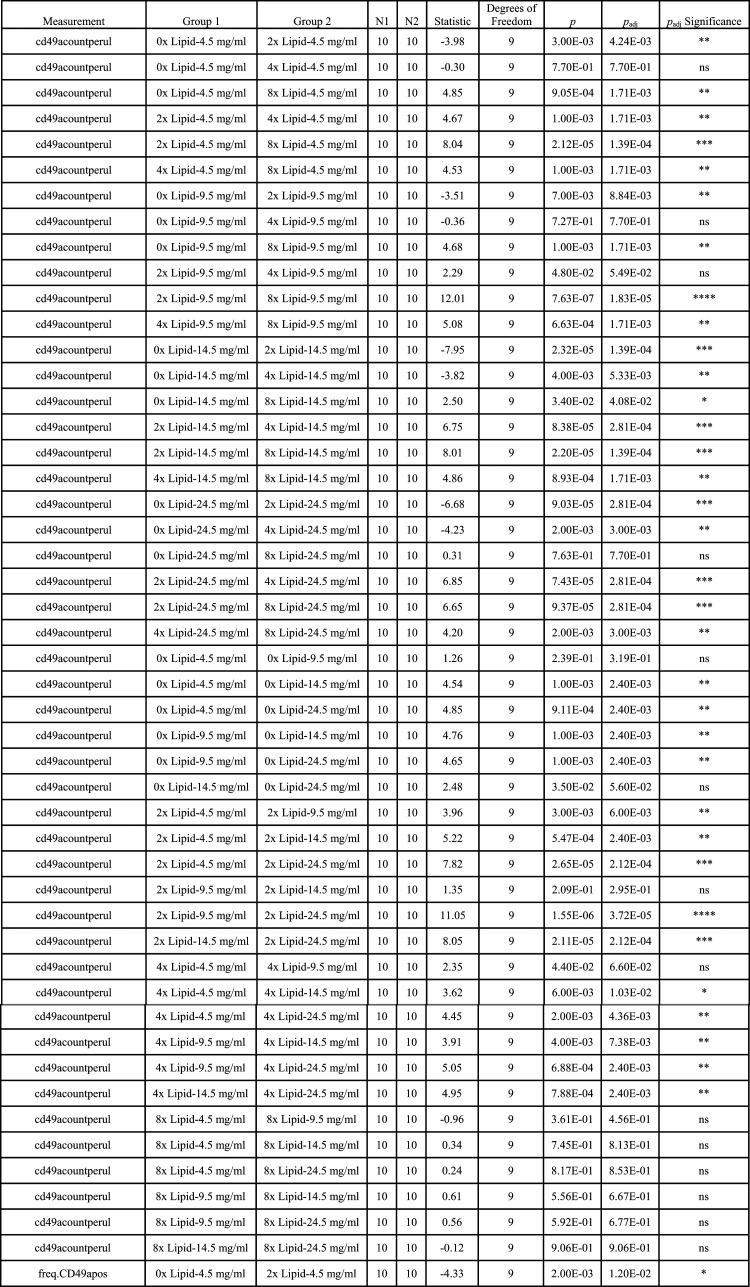

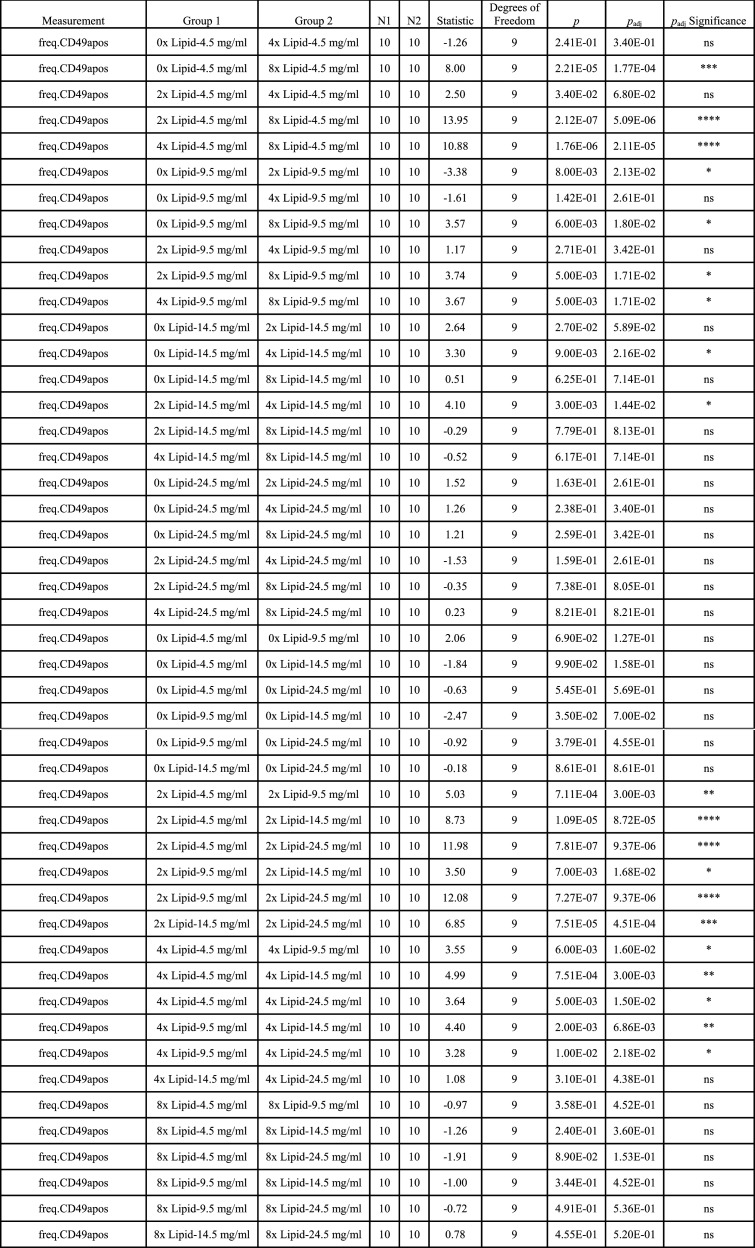

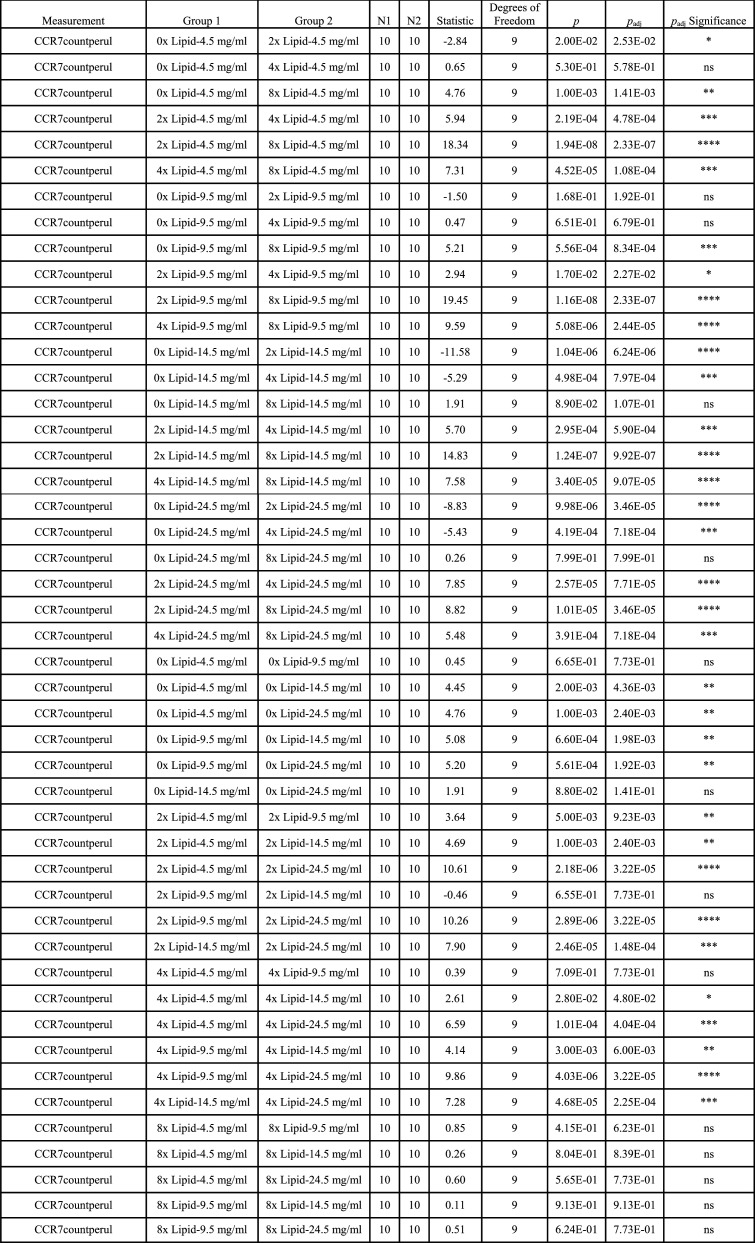

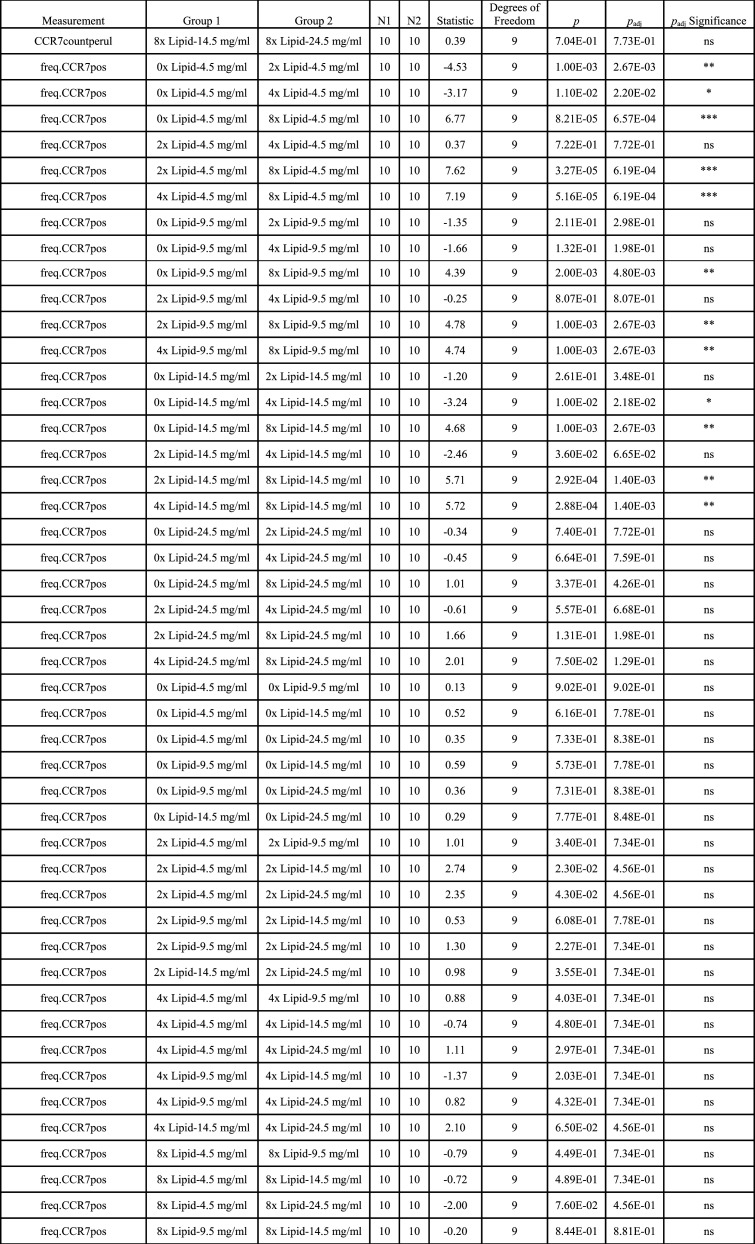

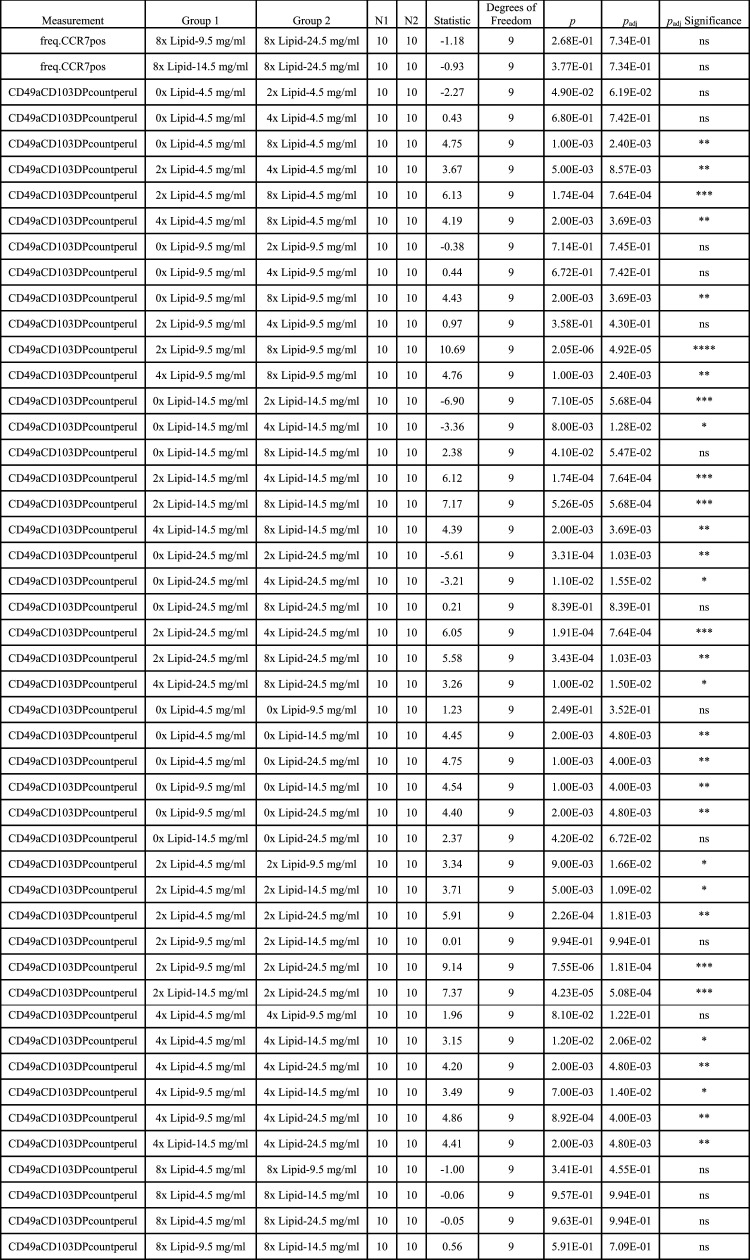

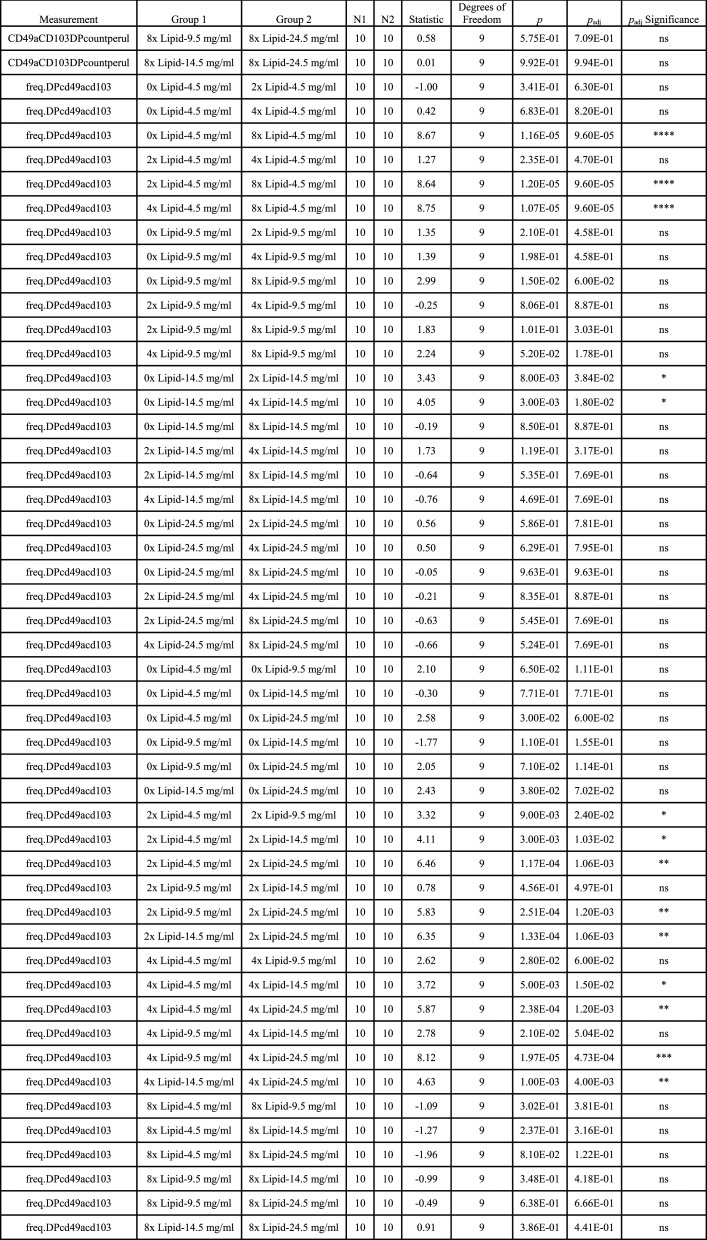
TABLE I. Statistical results for Figs. 3, 4, and 5

Group 1, the first group being compared; Group 2, the second group being compared; Measurement, what is being measured as the dependent variable; N1, number of animals in Group 1; N2, number of animals in Group 2; Statistic: *t* statistic. *p*_adj_ significance: ns: not significant, *p < 0.05, **p < 0.01, ***p < 0.001, ****p < 0.0001.

**FIGURE 5.**
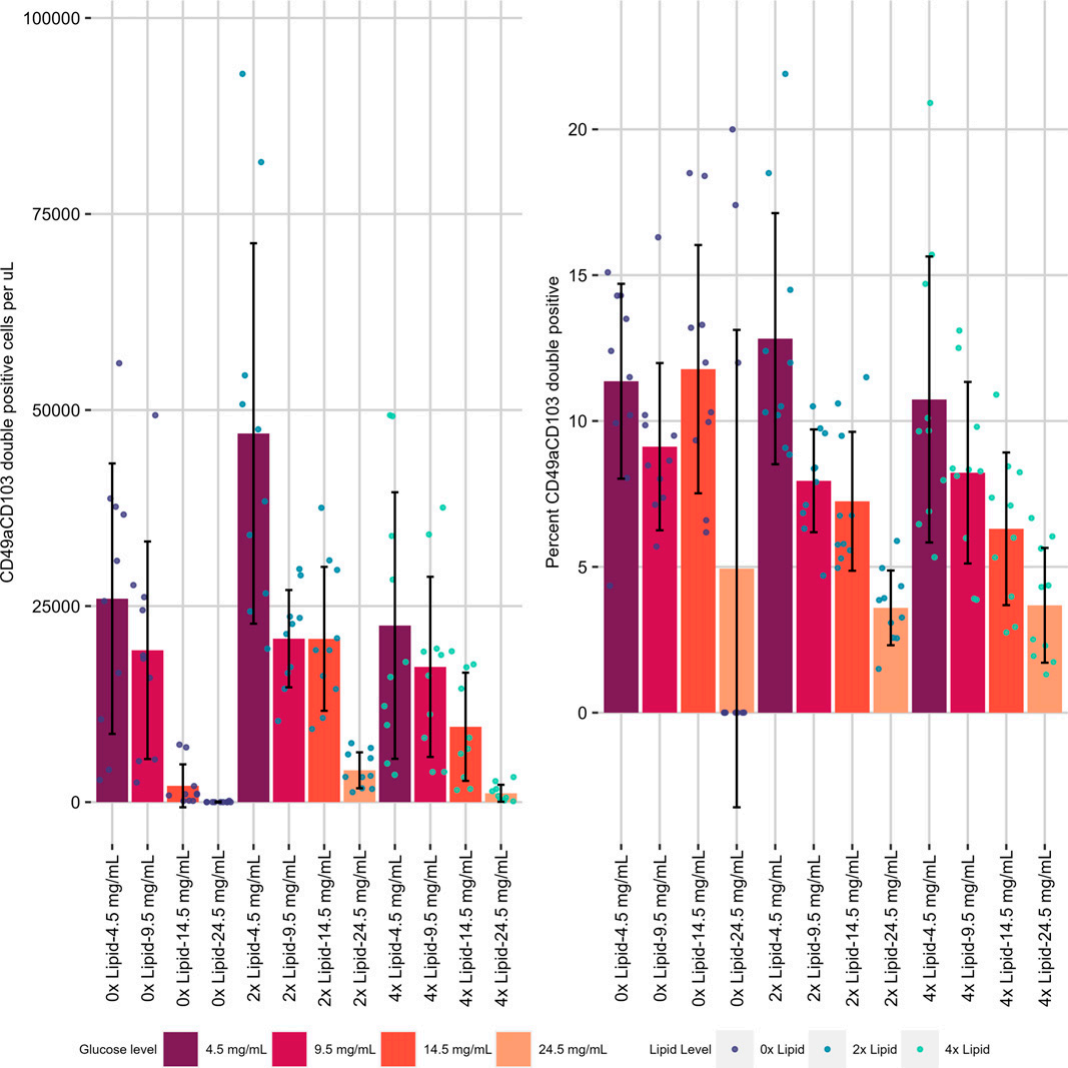
Metabolite concentration impact on CD49a^+^CD103^+^ T_RM_-like cell differentiation and maintenance in vitro. CD49a^+^CD103^+^ cells per microliter (**A**) and percent CD49a^+^ (**B**) were assessed in metabolite alteration assays where both lipid (0×–4×) and glucose (4.5–24.5 mg/mL) were altered after activation in the presence of IL-2 and cultured with IL-7 and TGF-β. Small circles represent individual data points. Data are generated from two independent experiments of *n* = 5 mice per experiment.

Looking at the effect of lipid supplementation at constant glucose levels on the differentiation of CD8 T cells in vitro, a contrasting pattern emerged: within each concentration of glucose a 2× lipid supplement increased the frequency of CD49a^+^ cells at the lower concentrations of glucose tested (4.5 and 9.5 mg/ml) (Fig. 3B, Table I). Further addition of lipid to 4× did not increase the frequency of CD49a^+^ cells. The absolute number of CD49a^+^ cells per volume peaked at 2× and decreased upon further addition of lipid for all concentrations of glucose tested. A similar but less drastic finding occurred for CCR7^+^ cells, but not for the frequency of positive cells (Fig. 4B, Table I). Patterns for CD49a^+^CD103^+^ cells were similar to those of CD49a SP (Fig. 5).

Taken together, these results imply that increasing glucose availability antagonizes the generation or maintenance of memory CD8 T cells independent of lipid supplementation and affects the expression of CD49a, at least in this assay. Furthermore, lipid supplementation is beneficial for CD49a expression up to a point but can be toxic at too high a level.

## Discussion

Using a genomic approach, we found memory T cell subsets in the lung exhibit distinct metabolic profiles. Furthermore, in vitro experiments conducted to drive T cells toward T_RM_-like surface phenotypes in the presence of varying metabolic substrates suggest metabolite availability can influence memory T cell differentiation and/or survival. In vivo metabolite availability may be caused by differences in local concentrations, the cell’s ability to transport metabolic substrates into the cell, or the expression of receptor(s) needed to take up these metabolites from outside the cell.

Differential expression, pathway, and metabolic modeling of our RNAseq data led to the hypothesis that lung T_RM_s have distinct metabolic programming compared with circulating cells. Using both functional and descriptive methods, we produced a dataset for a variety of cell functions, including mitochondrial profiling and nutrient uptake at both 14 and 60+ dpi. Integrating these results, what seems clear is that DP and CD49a SP, although different from each other in a few assays, share a similar pattern. This pattern is distinct from DN T cells, a mixed population consisting mostly of T cells with the phenotypes of effector memory and some central memory (7). The methods employed suggest DP and CD49a SP both take up more fatty acids, store more neutral lipids, and take up less glucose than DN cells at 14 dpi. At later memory time points, DP and CD49a SP diverge: DP cells have more mitochondrial mass and take up less glucose and more fatty acid than CD49a SP. Generally, memory cells have a higher SRC than effector cells, suggesting one possible advantage is to convey SRC (30). An increased SRC in DP, but not CD49a SP, could imply different roles in memory and effector responses. This should be cautiously interpreted because this SRC was not directly measured in this study, meriting further studies to validate this conclusion.

These findings are consistent with the interpretation that DP cells exhibit a more typical “resting” memory phenotype, and that CD49a SP could represent a more “activated” cell type. We have previously reported that the CD49a SP subset has a number of more effector-like features, such as having a higher frequency of Granzyme B and perforin DP cells, making them poised for cell killing (7).

The metabolic profile of an individual T cell, as well as a T cell subset, is important functionally in differentiation and effector function. Indeed, this is a leading hypothesis as to why some T cells become T_RM_s and some do not (29). The functions of these subsets during a subsequent infection may in part be achieved through the availability of different nutrients: if T_RM_s depend mostly on the uptake of FFAs, whereas naive and effector T cells depend more on glucose uptake, both may be able to perform their functions without competing for energy resources. Furthermore, these profiles can directly impact the cell signaling the cells are receiving. It is known that altering the ability of naive CD8 T cells to take up glucose directly impacts their likelihood of becoming memory cells such that the most glucose-starved T cells become memory cells (31). Having a metabolic profile that, at baseline, is not dependent on glucose may ensure that the internal cell signaling conferring this memory phenotype continues to do so in a positive feedback manner. TCR stimulation is directly related to the specificity of binding and the abundance of a TCR’s peptide:MHC complex. Others have shown that T cells take up more glucose when more activated (32). Having an abundance of high-affinity interactions with peptide:MHC complexes may cause a hyper-local effect of glucose starvation. The same group demonstrated that decreased availability of glucose led to increased memory CD8 T cell populations. A lipid-centric metabolism by memory T cells may assist naive T cells to respond to infection because they do not compete with other memory cells for glucose, making it less likely that memory T cells will starve out the naive responses. This may support eliciting a more diverse group of memory T cells at the end of an infection, which would increase the likelihood of a productive immune response upon subsequent heterologous infection. An important caveat to our interpretation is that NBDG does not always correlate with glucose uptake, even though it is a glucose analogue itself. As a result, more research must be done to confirm these conclusions.

The causal effect of metabolic factors on CD8 T cell differentiation has been studied, but never in the context of T_RM_ differentiation or in consideration of different T_RM_ subsets. In short, we found glucose to be detrimental to the in vitro–driven expression of T_RM_ marker CD49a in a linear manner, whereas we found increasing lipid beneficial, to a point. Conversely, glucose did not affect the expression of central memory marker CCR7, whereas the effect of lipids was similar for both CD49a and CCR7. Optimal concentrations may exist that increase or decrease the expression of these markers. We believe these findings have direct implications for the creation of therapies such as CAR-T cells, which necessitate ex vivo culture conditions. For example, conditions could be optimized for the creation of T cells with a T_RM_-like phenotype as observed among TILs associated with positive outcomes (33–36). Future studies should assess this possibility.

## Supplementary Material

Supplemental Figures 1 (PDF)

Supplemental Table I (XLSX)
