# Supplementary material for: Mouse Memory CD8 T Cell Subsets Defined by Tissue-Resident Memory Integrin Expression Exhibit Distinct Metabolic Profiles
Source: Immunohorizons. 2023 Oct 19;7(10):652–69. doi: 10.4049/immunohorizons.2300040 (PMC10615656; doi:10.4049/immunohorizons.2300040)
Supplement: Supplemental Figures 1 (PDF) [file IH_2300040_Supplemental_1.pdf]

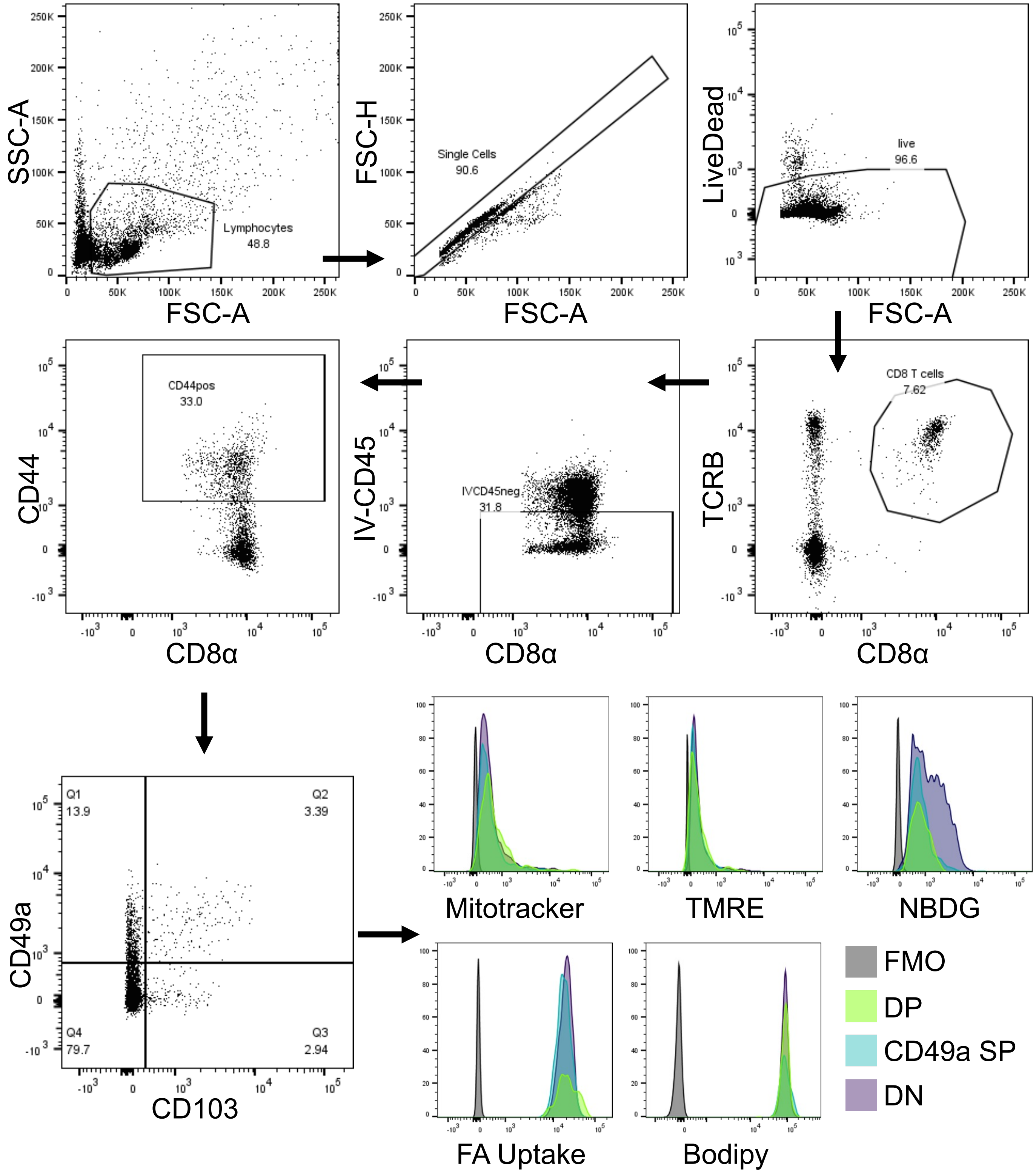

Supplementary Figure 1: Gating scheme and representative fluorescent dye staining for flow cytometry assays pictured in Figure 2.

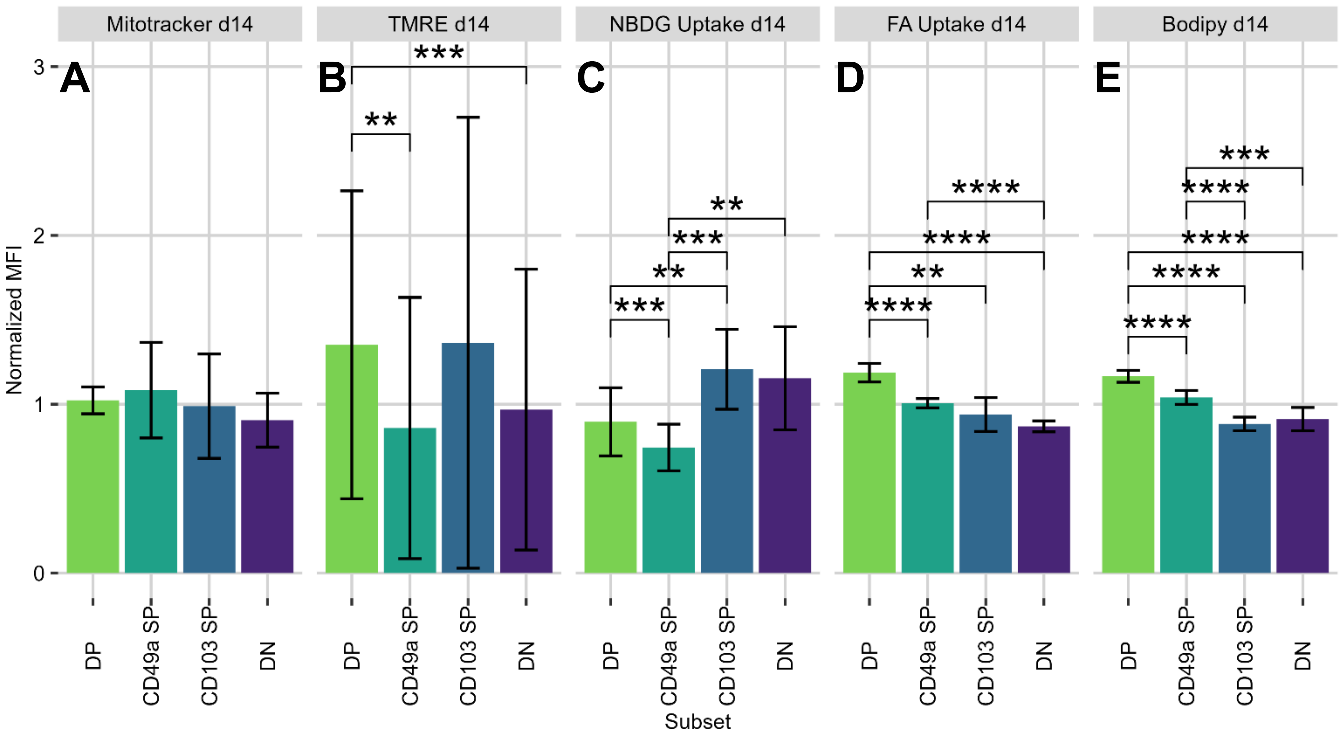

Supplementary Figure 2: Normalized mouse bronchoalveolar lavage non-intravenously labelled CD44^{pos} CD8 T cell MFIs from flow cytometric assays. *P < 0.05, **P < 0.01, ***P < 0.001, ****P < 0.0001. DP (Double Positive, CD49a^{pos}CD103^{pos}); CD49aSP (CD49a Single Positive, CD49a^{pos}CD103^{neg}); CD103SP (CD103 Single Positive, CD49a^{neg}CD103^{pos}); DN (Double Negative, CD49a^{neg}CD103^{neg}). Data were generated from two independent experiments of 4-10 mice each.

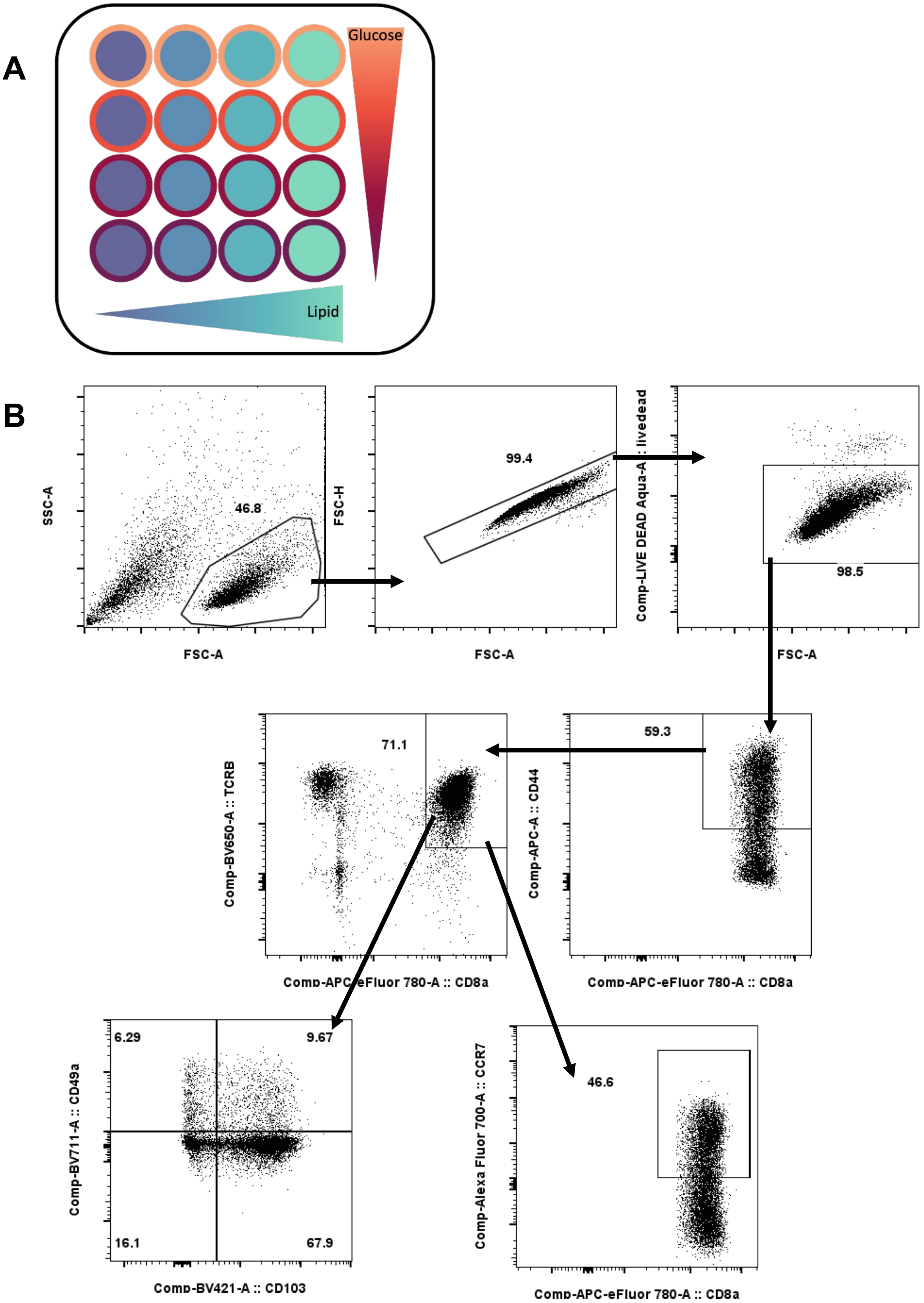

Supplementary Figure 3. Assay layout for T cell differentiation studies: 16 combinations (4 glucose treatment groups, 4 lipid treatment groups permuted) were assayed. Glucose groups included 4.5, 9.5, 14.5, and 24.5 mg/mL. Lipid groups included 0x, 2x, 4x, and 8x recommended concentrations (A). Gating scheme for differentiation studies (B).

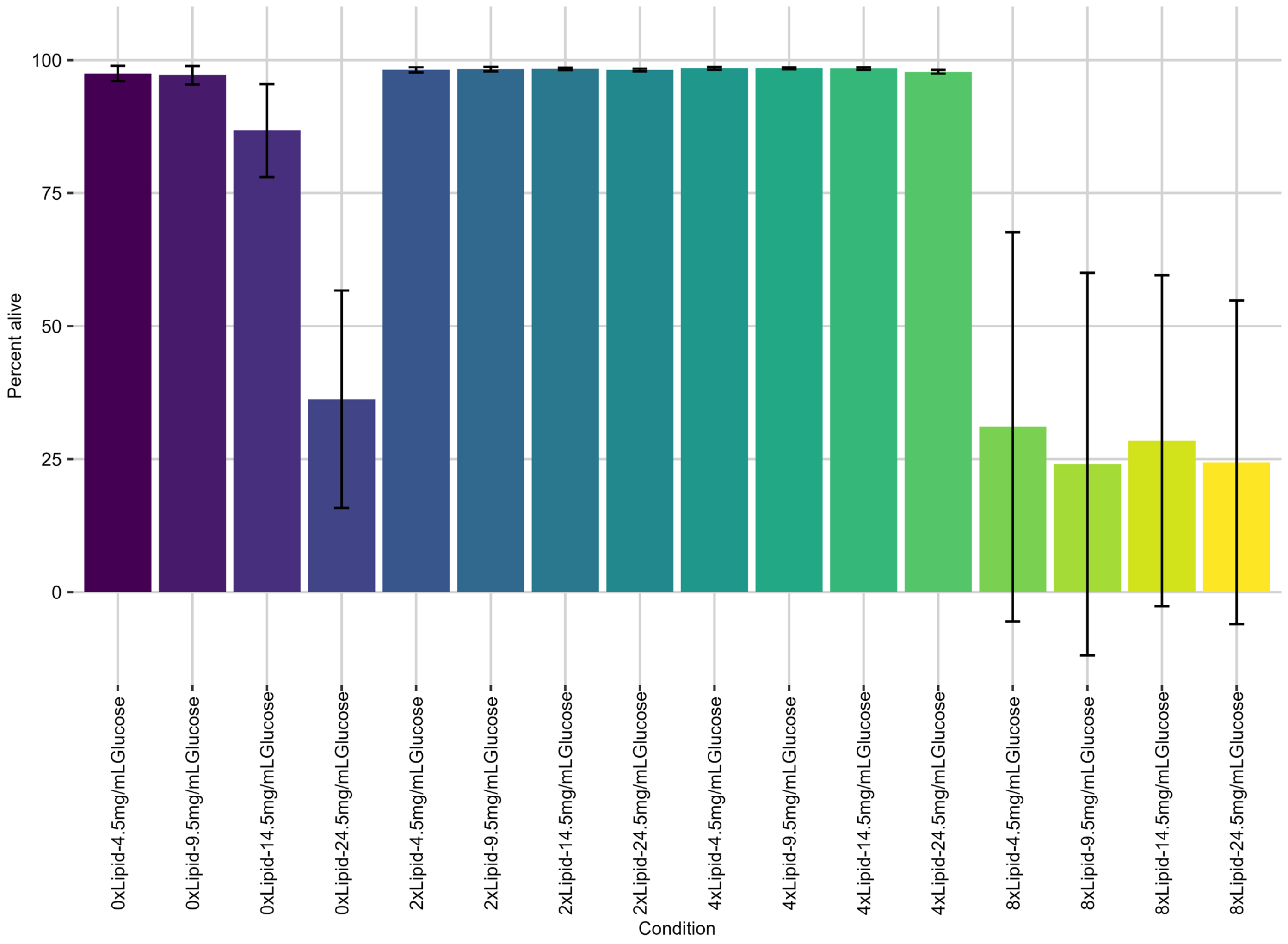

Supplementary Figure 4. Metabolite concentration impact on T cell viability *in vitro*. Percent alive in metabolite alteration assays where both lipid and glucose were altered. Data were generated from two independent experiments of 5 mice each.
